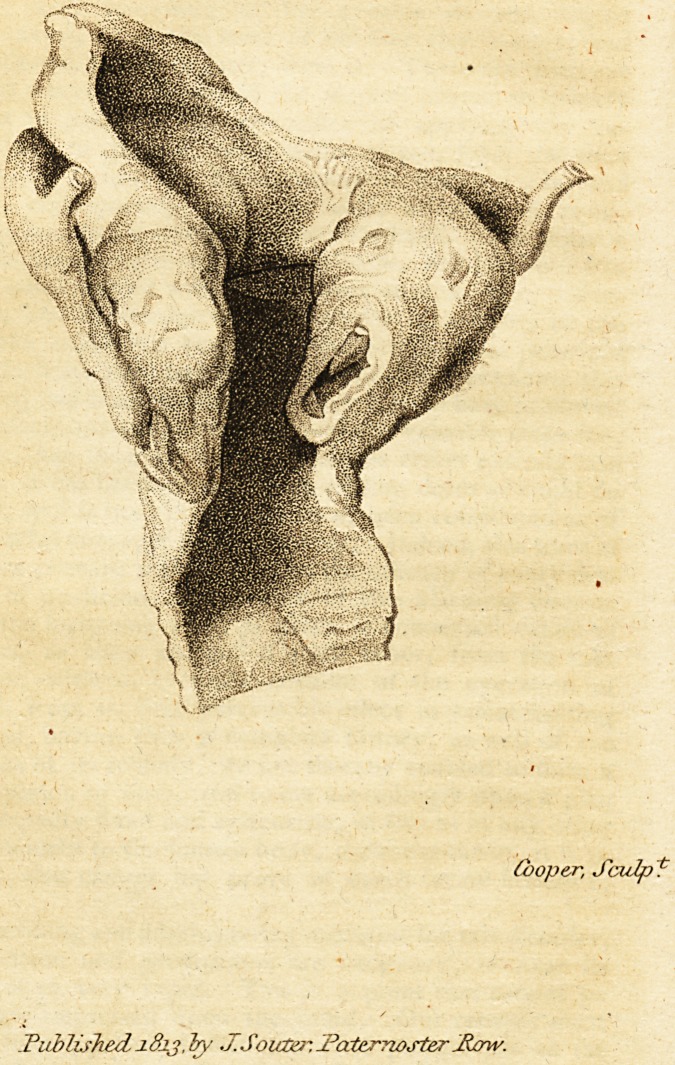# Mr. Shillito on an Inflammatory Affection of the Trachea

**Published:** 1813-02

**Authors:** Charles Shillito

**Affiliations:** Putney


*To the Editors of the Medical and Physical Journal.*

GENTLEMEN,

IN the last volume published by the Society for the improvement of Medical and Chirurgical Knowledge, three cases are given, by an eminent physician, of inflammatory affections of the windpipe. Two cases of the same disease have come within my observation, which I submit to you for insertion in your Journal, as it affords an opportunity of more extensively circulating his valuable observations on the most adviseable practice in those too-generally-fatal attacks.

The first case occurred at Horsham Barracks in the year 1809; and the following, as far as my recollection goes, assisted by some notes, are the particulars:—John Abrahart, a private soldier in the West Essex regiment of militia, about twenty-three years of age, of a rigid fibre and inflammatory diathesis, applied to me under the following circumstances. For two or three days he had been troubled with a slight

---

dry dry cough; he had also been occasionally chilly, and considered himself as having a common cold. Immediately previous to his coming to me, he had been attending divine service in the barrack-yard, where, he observed to me, his breathing had become so noisy as to attract the notice of his comrades near him, and he determined upon coming to the hospital. He had no acute pain, but a sense of uneasiness in the windpipe; his tongue was white, and the beat of his pulse quick and contracted; his breathing appeared to him to be accompanied by that peculiar shrill wheezing which occurs in croup; upon inspecting the fauces, neither the uvula or tonsils were altered from their natural appearance. He was immediately largely bled, had a saline purgative given him, and was ordered to bed. A few hours afterwards I saw him again, when he was laboring under much the same symptoms, but expectorated with his cough some viscid mucus. The blood taken away had strong marks of inflammation; and, as his pulse, although softer, was still too quick, he was bled a second time, ordered an antimonial medicine, and a large blister to the throat. The blood drawn the second time had not the cup-like contraction or buffy coat, but still the disease gained ground. He had more chills, was extremely restless, his respiration still more laborious, his countenance much dejected, and his articulation very thick. After any paroxysm of coughing, some ropy mucus was expectorated, but with difficulty. Ipecacuanha was given so as to produce vomiting, but without any material relief. Symptoms of suffocation continued to increase upon him; he had cold sweats; his pulse became small, tremulous, and intermitting; he was still sensible, and his countenance shewed every expression of horror, and had a dark or rather leaden hue. About thirty-four hours from his admission into the hospital, he died.

The next morning, with the assistance of Mr. Foaker, and Mr. John Dennis, at that time the assistant surgeons of the regiment, I examined the body. The lungs appeared quite sound; the windpipe, upon being detached, was found to be choaked up, and an abscess was discovered in the posterior part of the larynx, immediately under the membrane. Upon opening it with the point of the scalpel, as much pus issued out as would fill a common-sized tea-spoon. The membrane of the larynx throughout, and the epiglottis, seemed to be a little thickened, and some tenacious mucus was still adhering to it, but in small quantity. I had considered the case as a common one of croup, and, as the appearance of the windpipe upon dissection was unexpected and new to me, I wished to have it preserved, and sent it to

Dr.

Dr. Hooper, who very kindly gave it a place in his museum in Cork-street, where I believe it now is.*

During the last Spring I attended a carter in this neighbourhood, under nearly the same symptoms as the former, but less urgent. This patient was a thin puny lad, about twenty years of age, of a lax and delicate fibre, but predisposed to inflammatory attacks. The disease not having given way to bleeding (which in this case was but once had recourse to) and the usual antiphlogistic remedies, his pulse became quick and small, he had frequent cough and expectorated mucus, his respiration was laborious and sonorous, and he complained throughout the attack of much uneasiness in the windpipe, and desired to be kept propped up in bed, as being the most easy position to his breathing; his difficulty in which respect was becoming still more distressing, when he had a fit of coughing which lasted for several minutes, during which he spat up, besides the mucus which he had been before in the habit of doing, a small quantity of decided pus, and his breathing became at once easy, and he very soon got quite well.

I saw him soon after this unexpected relief, and considered the quantity of expectorated pus as being about half a tea-spoonful. No appearance of fullness or inflammation was discovered, in any stage of this attack, in either the tonsils or uvula; and, after much attention to the case at the time, I had not the smallest doubt of there having been a suppurative process in the windpipe. It may be right to mention, that, just previous to this illness, he had been pressed by a waggon against some paling, but which did not seem to have hurt him beyond a little temporary soreness of the muscles of the thorax. After his recovery, he went out to work during a keen easterly wind, and had an inflammatory attack upon his lungs, which he neglected until suppuration had taken place, and from which he eventually died.

Besides the three cases alluded to in the Medical and Chirurgical Transactions, where there was no peculiar or sonorous breathing, two cases of death from suffocation, where the disease was rapid in its progress, attended with difficult and noisy respiration, and pus found upon dissection in either the epiglottis or trachea, are given in your Journal.†

---

* We are favored with a Drawing from this valuable specimen, from which an Engraving will be made, and presented to our readers in the next Number of our Journal.—Editors.

† Numbers 78 and 139.

From

From the nature of the disease, which appeared after death in the case of my patient (Abrahart), it is not unreasonable to think that the abscess might have been accidentally opened in the operation of bronchotomy; or that it might have allowed the action of the lungs to continue until it spontaneously broke, as in the latter case. I certainly regret bronchotomy was not tried, particularly as Dr. Bailey, after the three fatal cases he has given in the work before-mentioned, observes, "As the inflammation in this disease is phlegmonic, it may be adviseable, at the very beginning of the attack, to take so much blood from the arm at once as to produce fainting. It is possible that benefit may be derived from this measure, although large bleeding in the common way was of no use. Opiates likewise might probably be employed with advantage to remove the spasm of the glottis, which certainly has some share in producing the difficulty of breathing, more especially where there are occasional feelings of suffocation. If no substantial advantage is produced by this plan in thirty hours, it might be adviseable to perform the operation of bronchotomy at the upper part of the trachea, just under the thyroid gland. This operation would probably enable the patient to breathe till the inflammation in the larynx, more especially at the aperture of the glottis, had time to subside."

As there is such good authority for hoping that, even in the inflammatory state, the operation might enable the patient to breathe until the inflammation subsides, in the suppurative stage, as in the first case I have given, where death could be satisfactorily traced to no other cause than the mechanical obstruction of an abscess preventing that ingress of air to the lungs which seems necessary to support life, and from which every muscle seemed to be unnerved, and indeed the whole system in the most complete state of exhaustion, it does not seem irrational to suppose that an opening in the windpipe, while the vital powers are yet acting, might so far resuscitate and carry on life, as to give nature time to terminate the disease, and the frame so far to rally as to afford some chance of its being able to get rid of any matter that might fall into the bronchia.

Cases of croup, even in infants, are at all times in their nature most distressing, and, when a disease so much resembling it occurs in an adult, where the sufferer until a very short period of his dissolution, is sensible of his misery, and imploring that relief from increasing suffocation which there has been hitherto a difficulty in giving, the feelings of his friends must naturally be strongly acted upon. Under such circumstances, it cannot be doubted that his medical attendants would anxiously urge, and the patient readily submit, to an operation operation which is allowed on such eminent authority as that I have before quoted, to afford such a reasonable chance of success, as to justify its being made a trial of in so fatal a disease. I am, Gentlemen,

*Putney,* Your obedient Servant,

*Jan.* 5, 1813. CHARLES SHILLITO.

## Figures and Tables

**Figure f1:**